# Nanostrip-Induced High Tunability Multipolar Fano Resonances in a Au Ring-Strip Nanosystem

**DOI:** 10.3390/nano8080568

**Published:** 2018-07-25

**Authors:** Zao Yi, Xin Li, Xibin Xu, Xifang Chen, Xin Ye, Yong Yi, Tao Duan, Yongjian Tang, Jiangwei Liu, Yougen Yi

**Affiliations:** 1 Joint Laboratory for Extreme Conditions Matter Properties, Southwest University of Science and Technology, Mianyang 621010, China; yizaomy@swust.edu.cn (Z.Y.); lixin1010106@yeah.net (X.L.); chenxifang1988@yeah.net (X.C.); twcsu2013@csu.edu.cn (Y.Y.); myyz1984@csu.edu.cn (T.D.); tangyongjian2000@yeah.net (Y.T.); 2 Sichuan Civil-Military Integration Institute, Mianyang 621010, China; 3 Research Center of Laser Fusion, China Academy of Engineering Physics, Mianyang 621900, China; dodolong@csu.edu.cn; 4 School of Energy Science and Engineering, Central South University, Changsha 410083, China; 5 College of Physics and Electronics, Central South University, Changsha 410083, China

**Keywords:** Au ring-strip nanosystem, multipolar Fano resonances, surface plasmon resonances, FDTD method

## Abstract

Surface plasmon resonances of a Au ring-strip nanosystem with tunable multipolar Fano resonances have been investigated based on the finite-difference time-domain (FDTD) method. Abundant plasmon properties of a Au ring-strip nanosystem can be obtained on the basis of the unique electronic properties of different geometry parameters. In our research models, these multipolar Fano resonances are induced and can be tuned independently by changing the geometry parameters of the Au ring-strip nanosystem. Complex electric field distributions excited by the Au ring-strip nanosystem provide possibility to form dark plasmonic modes. Multipolar Fano resonances display strong light extinction in the Au ring-strip nanosystem, which can offer a new approach for an optical tunable filter, optical switching, and advanced biosensing.

## 1. Introduction

In recent years, due to the ability to control light at visible and near-infrared wavelengths, and thereby induce very large electric field enhancement near the metallic micro/nanostructure surfaces, localized surface plasmon resonance (LSPR) of the metallic micro/nanostructure has been studied intensively [1,2,3,4,5]. LSPR has intriguing applications such as subwavelength waveguiding [6], biosensing [7], surface-enhanced Raman spectroscopy (SERS) [8], photovoltaic devices [9], and optical traps [10]. Recently, Fano resonances which arise from the interaction between bright and dark plasmonic modes are an interesting result of electromagnetic coupling in metallic micro/nanostructure [11,12]. Originating from quantum mechanics, Fano resonances are a general physical phenomenon found in systems comprising a coupling between a broad continuum of energy levels and discrete states. Due to their sensitive characteristic, Fano resonances have attracted significant attentions and have been used for biochemical sensing [13], second harmonic generation [14], SERS [15], nonlinear optics [16], and so on.

As Fano resonances arise from the coupling of micro/nanostructures, they are sensitive to the changes in geometry parameters of the micro/nanostructures: small perturbations can induce dramatic resonance or lineshape shifts. Plasmonic Fano resonance has been reported in a large number of metallic micro/nanostructure systems, such as nanorod complexes [17], core-shell nanoparticles [18], ring-disk nanostructures [19], oligomer clusters [20], nanoholes [21], and so on. The intensity and position of Fano resonances are mainly tuned by coupling with bright and dark plasmonic modes, which can be determined by the parameters of the nanostructure [22,23,24]. The effective achievement of the dark plasmonic modes in nanostructure is still an open question, because the dark plasmonic modes could not effectively couple with the electric field of the incident light once the wavelength of the excitation light is much greater than the size of the nanostructure. Dark plasmonic modes can be induced in large nanostructures because of the phase retardation effect. However, those dark plasmonic modes were usually excited by an electric field with grazing light, which hampers practical applications because most of these applications depend on the excitation of resonances by light at normal incidence [25,26,27]. Up to now, it is still a very large challenge to design nanostructures exhibiting tunable plasmonic Fano resonances at visible and near-infrared wavelengthss for their practical applications.

The multiple Fano resonances have been reported in previous literatures [28,29], but in the normal case, the multiple dark modes are simultaneously excited. Inspired by the experimental work of Lee et al. [30], in our research work, we designed Au ring-strip nanstructures to study the plasmonic multipolar Fano resonances. Compared with the nanostructure with a nanorod dimer and nanoring proposed by Lee et al. the multiple Fano resonances of our designed nanostructure are easier to be adjusted to the visible and near infrared by regulating the structural parameters. A variety of geometric parameters of Au ring-strip nanosystem were designed to regulate and control the plasmonic Fano resonances. Unique electronic and optical properties will induce new plasmonic Fano resonance properties in the Au ring-strip nanosystem. We hope the Au ring-strip nanosystem will provide new applications in surface-enhanced spectroscopy and optical switches.

## 2. Theoretical Methods

**Figure 1:**
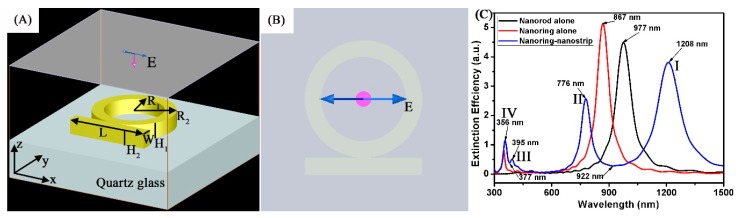
(**A**,**B**) Schematic illustration of the Au ring-strip nanosystem used in the simulation. (**C**) Extinction spectra of the Au nanostrip alone (black curve), the Au nanoring alone (red curve), and the Au ring-strip nanosystem (blue curve).

The plasmonic Fano resonances and near-field intensity distributions of the Au ring-strip nanosystem were simulated based on the finite-difference time-domain (FDTD) method. As shown in Figure 1A, the incident light wave polarized along the x-direction was illuminated from the SiO_2_ substrate, and it was perpendicular to the Au ring-strip nanosystem. The perfectly-matched layer absorbing boundaries were chosen as simulated boundary conditions. The simulation region is 0.14 × 0.14 × 0.14 μm, and the cell size is 0.5 × 0.5 × 0.5 nm. In this study, the dielectric functions of the Au ring-strip nanosystem were described using the experimental data of Johnson and Christy [31]. The refractive index of surrounding medium was set to be 1.0. The dielectric functions of the SiO_2_ were described by Palik [32]. Figure 1A,B display the detailed schematic of the simulated nanostructures. The structural parameters of the Au nanoring (outer radius (R_2_), inner radius (R_1_), and thickness (H_1_)) and nanostrip (length (L), width (W) and thickness (H_2_)) are shown in Figure 1A. In our research, we fixed the same thickness of H_1_ = H_2_ = 10 nm.

## 3. Results and Discussion

Figure 1C shows the extinction spectra of different models. In this case, for the Au nanoring, R_2_ and R_1_ are 30 nm and 20 nm, respectively. For the Au nanostrip, L is 60 nm and W is 10 nm. The polarization direction of the plane wave is parallel to the x-axis. As depicted in Figure 1C (the black curve), for the incident light wave polarized along the long axis of the Au nanostrip, the extinction spectra has a longitudinal dipole resonance peak at 977 nm [33]. As shown by the red curve, for a single Au nanoring, the spectrum displays the transverse dipole bonding mode at 867 nm, which is a result of the symmetric coupling between the internal and external surfaces of the Au nanoring dipolar modes [34]. As shown by the blue curve, for Au ring-strip nanosystem, the extinction spectra shows very different absorption peaks. As shown in Figure 1A,B, the Au nanoring and nanostrip are combined into a symmetric ring-strip composite nanostructure. The extinction spectra of Au ring-strip nanosystem displays four optical resonances. In addition to a transverse dipole bonding mode I (the bright plasmonic mode) at 1208 nm, higher resonance modes of II (the dark plasmonic mode), III (the dark plasmonic mode), and IV (the bright plasmonic mode) are observed at 776 nm, 395 nm, and 356 nm, respectively. This may be because polarized light can excite both symmetric and anti-symmetric modes simultaneously for the composite structure. We define the quality factor (Q) as the resonant wavelength over the width of the resonance. The Q factor of I, II, III, and IV modes are 8.9, 14.4, 20.4, and 38.3, respectively. As shown by the blue curve, two apparent minimum resonance peaks at the resonance wavelength of 922 nm and 377 nm were found, where the dark plasmonic mode and the bright plasmonic mode are spectrally overlapped. The excellent dark plasmonic modes excited in the Au ring-strip nanosystem can make them good choices to interact well with the bright plasmonic modes, where the plasmonic Fano resonances can be easily tuned.

**Figure 2:**
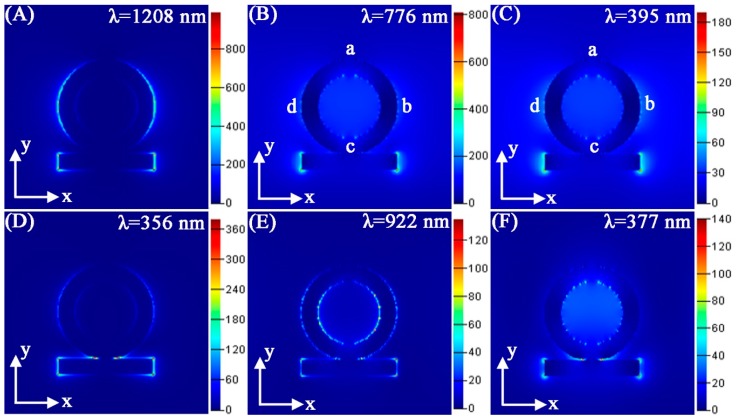
(**A**–**F**) Calculated electric field |E_2_|/|E_0_^2^| of the Au ring-strip nanosystem at different resonance wavelengths. The structure parameters are as follows: W = 10 nm, R_1_ = 20 nm; R_2_ = 30 nm; L = 60 nm. The color bars in the logarithmic scale for electric enhancement are shown (the units are (V/m)^2^).

Figure 2 displays the field distributions of Au ring-strip nanosystem surfaces at four strong absorption peaks located at 1208, 776, 395, and 356 nm, and two minimum absorption peaks located at 922 and 377 nm. The field distributions in Figure 2A display local field of the resonance peak at 1208 nm. Here, the dipole-dominant field distributions are along the x-polarization. The field distributions are around the outer surface of the Au nanoring and two ends of the nanostrip. As shown in Figure 2B, the strong field distributions mainly appear at both ends of the nanostrip. The weak charge distributions on the Au nanoring exhibit four nodes, which implies quadrupole plasmon modes there. Therefore, the resonance at 776 nm is the dark plasmonic resonance mode. As shown in Figure 2C, the electric field distributions are similar to that of Figure 2B, indicating that they are also the dark field mode. As shown in Figure 2D, the strong electric field distributions are concentrated at the junction between the nanoring and nanostrip. According to an analysis of the absorption peaks at 1208, 776, 395, and 356 nm, minimum resonances at 922, and 377 nm provide the possibility to produce multipolar Fano resonances. As shown in Figure 2E, the field distributions at 922 nm present a very complex scene. As we know, plasmon oscillations at Fano resonance can be dispersed to localized regions. Therefore, bright plasmonic mode at 1208 nm and dark plasmonic mode at 776 nm can produce the Fano resonance at 922 nm. As shown in Figure 2F, the multipolar field distributions mainly appear around the inner surface of nanoring. Due to the coupling of bright and dark plasmonic modes, the complex field distributions were generated.

**Figure 3:**
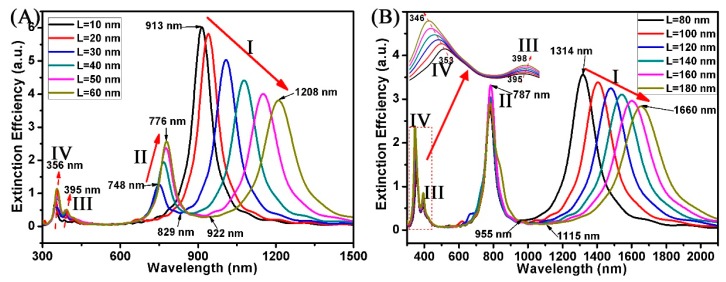
(**A**,**B**) Extinction spectra of the Au ring-strip nanosystem with different Au nanostrip length (L). The radius of the Au nanoring are unchanged (R_2_ = 30 nm; R_1_ = 20 nm). W of the Au nanostrip remains unchanged (W = 10 nm). L of the Au nanostrip increases from 10 to 180 nm.

As we know, the plasmon characteristics of Au nanorods are mainly affected by the longitudinal dipole modes [35,36]. In our study, we can also predict that the geometry parameters of Au nanostrips will also affect the plasmon characteristics of the Au ring-strip nanosystem. Figure 3 displays the extinction spectra of the ring-strip nanosystem with different L of Au nanostrip. In this case, the R_2_ and R_1_ of the Au nanoring are fixed at 30 nm and 20 nm, respectively. The W of the Au nanostrip is fixed at 10 nm. The L of the Au nanostrip varies from 10 to 180 nm. Due to the coupling of the nanoring and nanostrip, these nanostructures display four well-defined peaks at I, II, III, and IV. However, with the increase of the L of Au nanostrip, the extinction peaks of different modes show different changes. When the L changes from 10 to 180 nm, the dipole resonances of mode I red shift from 913 to 1660 nm. Additionally, the relative intensity ratio of dipole resonances reduces from 6.8 to 2.8. For Au nanorods, the dipole resonances red shift and relative intensity ratio of dipole resonances decreases with increasing aspect ratio [35,36]. In the Au ring-strip nanosystem, the charge assigned to the Au nanoring will increase with the increase of the L of nanostrip. This explains why the relative intensity ratio of resonances of mode II increases with the increase of the L of nanostrip, which increases from 1.2 to 3.3. When the L of the Au nanostrip varies from 10 to 180 nm, the resonances of mode II have red shift and the extinction efficiency has increased. The resonances of mode II red shift from 748 nm (L = 10 nm) to 787 nm (L = 80 nm). At the same time, the extinction efficiency increases from 1.2 to 2.7. When the L of the Au nanostrip varies from 80 to 180 nm, the resonances of mode III red shift from 395 to 398 nm, and the resonances of mode IV blue shift from 353 to 346 nm. The corresponding extinction spectra of the Au ring-strip nanosystem exhibit Fano resonances, where the Fano resonances red shift from 829 to 1115 nm with the L of nanostrip increasing from 20 to 180 nm. The spectrum modulations from visible and near infrared light are important for sensing.

**Figure 4:**
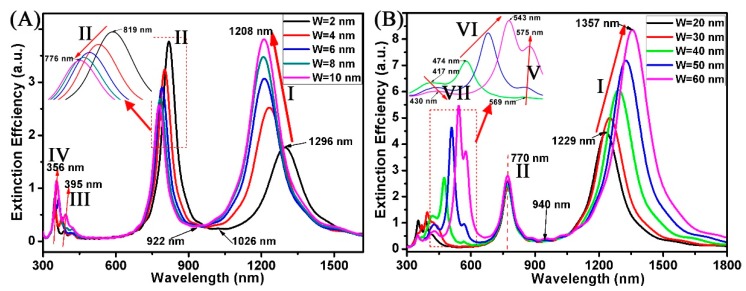
(**A**,**B**) Extinction spectra of the Au ring-strip nanosystem with different W of the Au nanostrip. The radius of the Au nanoring are unchanged (R_2_ = 30 nm; R_1_ = 20 nm). L of the Au nanostrip is unchanged (L = 60 nm). W of the Au nanostrip increases from 2 to 60 nm.

Figure 4 shows the extinction spectra of the Au ring-strip nanosystem with different W of Au nanostrip. In this case, the R_2_ and R_1_ of the Au nanoring are fixed at 20 nm, and 30 nm, respectively. The L of the Au nanostrip is fixed at 60 nm. The W of the Au nanostrip varies from 2 to 60 nm. Here, the W for simulation can be classified into two types of cases, one with the W of nanostrip greater than the fixed thickness of the nanoring (t, t = R_2_ −R_1_ = 10 nm), and the other smaller than it. When the W varies from 2 to 10 nm (W < t), as shown in Figure 4A, the resonances of mode I blue shift from 1296 to 1208 nm. The relative intensity ratio of resonances increases from 1.7 to 3.8. The reason is that the ratio of the surface atoms to total atoms in the nanostructure varies. When all other parameters are fixed, the ratio increases when the W of the Au nanostrip increases. As the W of the nanostrip varies from 2 to 10 nm, the resonances of mode II have blue shift and the extinction efficiency has decreased. The resonances of mode II blue shift from 819 nm (W = 2 nm) to 776 nm (W = 10 nm). At the same time, the extinction efficiency decreases from 3.8 to 2.6. When the W of the nanostrip increases, the electrons transferred to the Au nanoring will decrease. Therefore, the absorption peaks (mode II) blue shift and the intensity decreases. The Fano resonances blue shift from 1026 to 922 nm with the W of nanostrip increasing from 2 to 10 nm. As shown in Figure 4B, when the W of nanostrips is larger than 10 nm, variation trend of absorption peaks has distinct differences, and some new absorption peaks appeared in these curves. When the W of the nanostrip is 10 nm, the resonance of mode I has the largest blue shift (1208 nm) in all the curves. When the W varies from 20 to 60 nm (W > t), as shown in Figure 4B, the resonances of mode I have red shift and the extinction efficiency has increased. The resonances of mode I red shift from 1229 nm (W = 20 nm) to 1357 nm (W = 60 nm). At the same time, the extinction efficiency increases from 4.4 to 8.3. However, the resonances of mode II are maintained at 770 nm. This indicates that the nanostrip does not affect the resonances of mode II when the W of the nanostrip is greater than 10 nm. Since the peak shift of mode I and mode II is not significant, the Fano resonances are maintained at 922 nm. When the W of the nanostrip is greater than the thickness (t) of the nanoring, the Fano resonances are not suitable adjustable. When the W varies from 40 to 60 nm (W ≥ 2t), as shown in Figure 4B, some new multipolar absorption peaks (V, VI, and VII modes) appeared in these curves.

**Figure 5:**
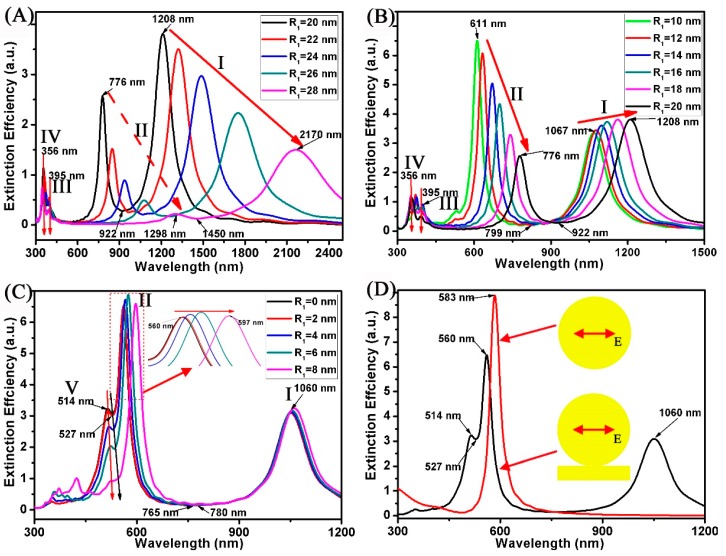
(**A**–**D**) Extinction spectra of the Au ring-strip nanosystem with different R_1_ of the Au nanoring. The outer radius of the Au nanoring are unchanged (R_2_ = 30 nm). L and W of the Au nanostrip are unchanged (L = 60 nm; W = 10 nm). R_1_ of the Au nanoring increases from 0 to 28 nm.

In our previous studies, the increase of nanoring radius can effectively promote the red shift of the absorption peak of the surface plasmon [37]. Figure 5 displays the extinction spectra of the Au ring-strip nanosystem with different R_1_ of Au nanoring. In this case, the R_2_ of the Au nanoring is fixed at 30 nm. The L and W of the Au nanostrip are fixed at 60 and 10 nm, respectively. The R_2_ of the Au nanoring vary from 0 to 28 nm. When the R_1_ varies from 20 to 28 nm, as shown in Figure 5A, the resonances of mode I and mode II red shift and the extinction efficiency is decreased, and the shift range of Fano resonances is very large, which red shift from 922 to 1450 nm. When the R_1_ varies from 10 to 20 nm, as shown in Figure 5B, the red shift range of resonances (mode I and mode II) is decreasing, which red shift from 1067 to 1208 nm and red shift from 611 to 776 nm. For mode II, the extinction efficiency is increased. When the R_1_ is 20 nm, and the thickness (t, t = R_2_ − R_1_) of nanoring is 10 nm, the extinction efficiency of the resonances (mode I) has a maximum value (3.8), and the red shift range of Fano resonances decreases, red shifting from 799 to 922 nm. When R_1_ varies from 0 to 8 nm, as shown in Figure 5C, the resonances of mode I are maintained at 1060 nm. For the resonances of mode II, the red shift range is from 560 to 597 nm. When R_1_ is 6 nm, the extinction efficiency of resonances (mode II) has a maximum value (6.9). Since the shift range of mode I and mode II is very small, the shift range of Fano resonances red shift from 765 to 780 nm only. Here, when the R_1_ is less than 8 nm, new additional absorption peaks (mode V) appear at 514 nm, which is a result of dipolar plasmon mode of nanodisk [38]. Here, when the R_1_ increases to 8 nm, the new Fano resonances at 527 nm gradually weaken until they disappear. For a single Au nanodisk, as shown by the red curve in Figure 5D, the spectrum shows a dipolar excitation at 583 nm only.

**Figure 6:**
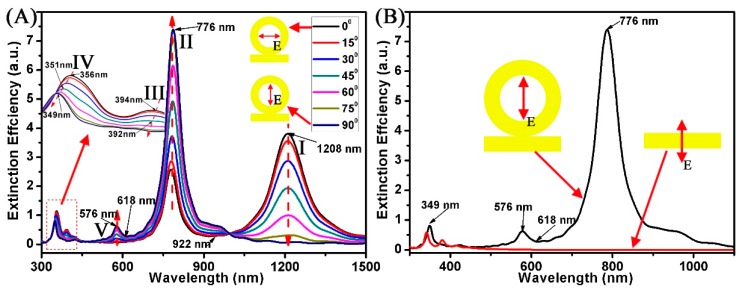
(**A**,**B**) Extinction spectra of the Au ring-strip nanosystem with different polarization direction (θ). Other parameters are unchanged (W = 10 nm; R_1_ = 20 nm; R_2_ = 30 nm; L = 60 nm). The θ increases from 0° to 90°.

The multipolar Fano resonances characteristics of the proposed nanostructure are also dependent on the polarization direction of the incident light [39,40]. Figure 6 displays the calculated extinction spectra of the Au ring-strip nanosystem with different angles θ (θ is the angle between the incident light and x-axis). The structural parameters are fixed (W = 10 nm; R_1_ = 20 nm; R_2_ = 30 nm; L = 60 nm). The θ changes from 0° to 90°. It can be found for Au ring-strip nanosystem with different θ between 15° and 75°, five SPR peaks appear in these curves. For instance, at θ = 45°, five SPR peaks appear at 351, 392, 576, 776, and 1208 nm, respectively. Here, these SPR resonances are designated as five modes (I, II, III, IV, and V modes). As shown in Figure 6, when the θ changes from 0° to 90°, the extinction efficiency of resonances (mode I) gradually decreases to zero. However, for the absorption peaks at 776 nm (mode II), the extinction efficiency increases from 2.6 to 7.4. When θ changes from 0° to 75°, the Fano resonances are maintained at 922 nm. However, when θ is 90°, the Fano resonance at 922 nm disappears. The reason is that the Fano resonances can be effectively excited by Ex component of incident light because of the strong interaction between the I and II modes. When θ is 90°, because only the Ey component exists, mode II can be effectively excited while mode I cannot be excited. When θ varies from 15° to 90°, new absorption peaks (mode V) appear at 576 nm. The imperfect Fano resonances at 618 nm are induced because of the interactions among the dipole mode and the quadrupole mode, which are induced by the Ex and Ey components of the incident light. The Fano resonances at 618 nm gradually weaken until they disappear with the decrease of θ, accompanyied with the process from a strong coupling to a small coupling between the excited dipole mode and the quadrupole mode. When the incident light is perpendicular to the long axis of the Au nanostrip (θ = 0°), as shown by the red curve in Figure 6B, the extinction spectra has a dipole resonance peak at 349 nm with weak intensity [40].

## 4. Conclusions

In summary, the combination of nanostrip in Au ring-strip nanosystems has been proved to be effective in adjusting the surface plasmon frequency. The extinction spectra of Au ring-strip nanosystems display different absorption modes of the plasmon compared with only the nanoring or nanostrip. The surface plasmon interaction at the interface between ring and disk strip can alter the SPR of the Au ring-strip nanosystems greatly. These extinction spectra of Au ring-strip nanosystems with different geometry parameters display the multipolar surface plasmons modes. By regulating geometry parameters, these Fano resonances have wide regulation from visible light to mid-infrared. Furthermore, simulation of Au ring-strip nanosystems with different polarization directions of incident light (θ) has also been considered in our study. The change of the polarization direction can control the generation and extinction of these Fano resonances. We hope that the Au ring-strip nanosystems can provide a new application for designing a plasmonic Fano switch and multiwavelength surface-enhanced spectroscopy.
